# Neoadjuvant Immunotherapy-Based Treatment Versus Chemotherapy Alone in Resectable Locally Advanced dMMR/MSI-H Gastric Cancer: A Real-World Study with Meta-Analysis

**DOI:** 10.3390/cancers18122017

**Published:** 2026-06-22

**Authors:** Huayang Pang, Yan Chen, Zhou Zhao, Zehua Chen, Menghua Yan, Bo Yi, Xiufeng Chen, Hao Sun

**Affiliations:** 1 Department of Gastrointestinal Cancer Center, Chongqing University Cancer Hospital, Chongqing 400030, China; panghy2031@163.com (H.P.); 15178913507@163.com (Y.C.); zhaozhou_med@163.com (Z.Z.); chenzehuamed@163.com (Z.C.); 18996369597@163.com (M.Y.); yb13786179533@163.com (B.Y.); 2 Chongqing Key Laboratory of Translational Research for Cancer Metastasis and Individualized Treatment, Chongqing University Cancer Hospital, Chongqing 400030, China

**Keywords:** locally advanced gastric cancer, mismatch repair deficient, microsatellite instability high, neoadjuvant treatment, pathological response outcomes, survival outcomes

## Abstract

**Background:** Evidence suggests dMMR/MSI-H gastric cancer patients respond better to immune checkpoint inhibitors (ICIs) than to chemotherapy, but recent trials have not consistently shown this benefit in subgroup analyses. It remains unclear whether improved the pathological response translates into survival benefit. This study compares pathological response and survival outcomes between neoadjuvant immunotherapy regimens (ICI monotherapy, ICI plus chemotherapy, or dual ICIs; group A) and chemotherapy alone (group B) in locally advanced dMMR/MSI-H gastric cancer. **Methods:** Between January 2020 and December 2025, 24 patients undergoing surgery were enrolled—14 in group A and 10 in group B. The primary endpoints were major pathological response (MPR), pathological complete response (pCR), event-free survival (EFS), and overall survival (OS). A meta-analysis integrating our cohort with external studies was conducted to strengthen the evidence base. **Results:** In our cohort, compared with group B, group A appeared to have higher rates of MPR (85.7% vs. 20%) and pCR (42.9% vs. 0%). EFS and OS in group A improved numerically (EFS: *p* = 0.10; OS: *p* = 0.12). Surgical outcomes and treatment-related adverse events were not different between the two groups. Pooled analyses implied consistent improvements in MPR (RR = 4.09) and pCR (RR = 5.38). Additionally, reconstructed individual survival data suggested that group A might have better EFS (*p* = 0.034) while OS (*p* = 0.890) showed little difference. **Conclusions:** Neoadjuvant immunotherapy-based regimens might show some enhancements in treatment response rates and EFS compared with chemotherapy alone, which may imply their therapeutic potential in this molecularly defined patient population.

## Simple Summary

Evidence suggests that patients with dMMR/MSI-H gastric cancer may have a better response to immune checkpoint inhibitors (ICIs) than to conventional chemotherapy. Yet several recent neoadjuvant trials have not consistently shown improved pathological responses with immunotherapy. Moreover, even if a certain level of pathological response is detected, it remains unclear whether such responses can lead to survival benefit. By combining real-world data from our center with previous studies, we found that neoadjuvant immunotherapy-based regimens (including ICI monotherapy, ICI combined with chemotherapy, and dual ICIs) may improve the major pathological response rate and pathological complete response rate. For long-term outcomes, immunotherapy-based treatment showed potential for improving event-free survival. However, the evidence for overall survival benefit remains inconclusive at this stage. Overall, these results suggested that neoadjuvant immunotherapy-based treatment shows potential clinical value in these patients, but larger-scale prospective studies with adequate follow-up time are urgently needed to better understand this issue.

## 1. Introduction

Gastric cancer ranks fifth among global malignant tumors in terms of both incidence and mortality, with the majority of cases diagnosed at the locally advanced stage [1]. For patients with locally advanced gastric cancer (LAGC), neoadjuvant chemotherapy has demonstrated efficacy in downstaging tumors and improving radical resection (R0 resection) rate and long-term survival [2]. Currently, in Europe and the United States, the FLOT (5-Fluorouracil, Leucovorin, Oxaliplatin and Docetaxel) regimen is the standard of care for neoadjuvant chemotherapy; by contrast, the SOX (S-1 and Oxaliplatin) and CAPOX (Capecitabine and Oxaliplatin) regimens are more widely adopted in Asian clinical practice [3]. Nevertheless, despite comprehensive perioperative management, the pathological complete response (pCR) rate in LAGC patients remains about only 6% to 16%, and the 5-year overall survival (OS) rate is below 50%, highlighting the urgent need for more-effective therapeutic strategies [4,5].

In LAGC, tumors with mismatch repair deficiency (dMMR) or microsatellite instability-high (MSI-H) status constitute approximately 3.9% to 12.4% of total cases [6]. These tumors generally show a favorable prognosis but respond poorly to conventional chemotherapy—sometimes even resulting in harm [7]. With the advent of immune checkpoint inhibitors (ICIs), especially PD-1 and PD-L1 inhibitors, the treatment landscape for such tumors has seen a turning point [8]. The INFINITY trial [9] reported a pCR rate of 60% among 15 LAGC dMMR/MSI-H patients who received neoadjuvant tremelimumab plus durvalumab, with a two-year OS rate of 92%. In China, the recently published NICE trial [10] observed an even higher pCR rate of 80% with neoadjuvant toripalimab plus chemotherapy in these patients; notably, no recurrence was observed during a median follow-up of 22.5 months.

However, although neoadjuvant immunotherapy-based regimens have shown promising activity in this patient population, the majority of supporting evidence derives from single-arm clinical trials or retrospective studies with limited follow-up durations [10,11,12]. Consequently, whether such regimens confer a definitive advantage over neoadjuvant chemotherapy alone remains unestablished. In the recently published phase III MATTERHORN trial [13], neoadjuvant durvalumab plus FLOT failed to demonstrate statistically significant improvements in pCR and event-free survival (EFS) rates compared with neoadjuvant FLOT alone in LAGC dMMR/MSI-H patients. Additionally, Raimondi et al. [7] designed individual patient data (IPD) meta-analyses to compare different perioperative treatment modalities in resectable dMMR/MSI-H LAGC patients. While neoadjuvant immunotherapy significantly increased both pCR and major pathological response (MPR) rates relative to neoadjuvant FLOT alone, no statistically significant differences were observed in 3-year EFS or 3-year OS rates. In Asia, a retrospective study conducted by Zhang et al. [14] reported numerically higher pCR and EFS rates with neoadjuvant immunotherapy-based regimens versus chemotherapy alone yet all comparisons lacked statistical significance (all *p* > 0.05).

Here, we retrospectively collected and reported our institutional data, aiming to compare the pathological response outcomes and long-term survival outcomes of neoadjuvant PD-(L)1 inhibitor with or without chemotherapy versus chemotherapy alone in patients who were diagnosed with resectable dMMR/MSI-H LAGC and received gastrectomy. In addition, to strengthen the evidence base for clinical decision-making in this context, we conducted a meta-analysis integrating data from the published literature with the data obtained in this study.

## 2. Material and Methods

### 2.1. Ethics Statement

This retrospective study was conducted in full compliance with the ethical principles of the Declaration of Helsinki and approved by the Research Ethics Committee of Chongqing University Cancer Hospital (Approval No.: CZLS2026237-A). To safeguard participant confidentiality, all medical records were anonymized and de-identified prior to analysis. Written informed consent was obtained from each patient before initiation of treatment.

### 2.2. Study Population

**Figure 1:**
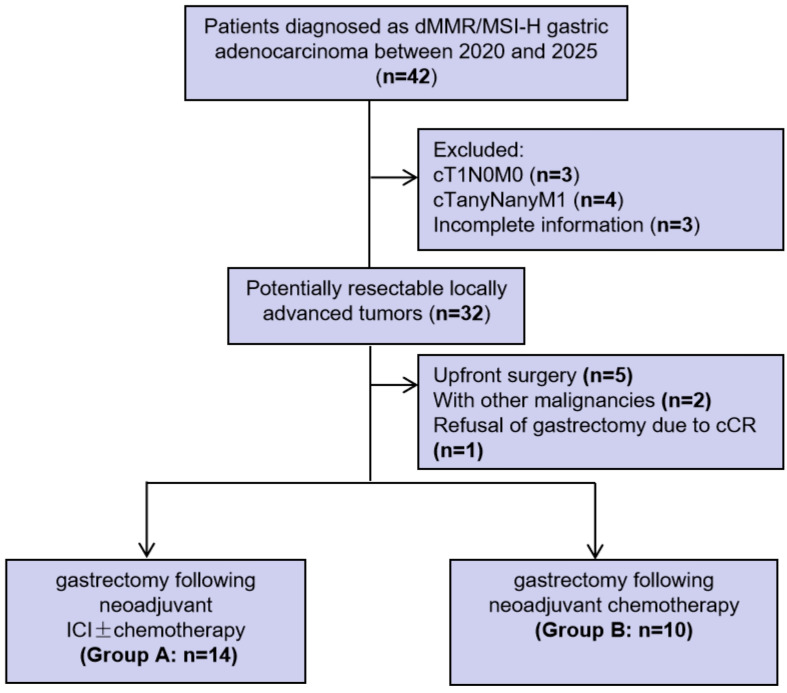
The flow chart of patients enrolled in this study.

Since 2020, our center has routinely assessed the dMMR/MSI-H status of newly diagnosed gastric cancer patients using tumor biopsy specimens. Herein, dMMR/MSI-H LAGC patients diagnosed and managed at our institution between January 2020 and December 2025 were consecutively enrolled (Figure 1). Inclusion criteria were: (1) imaging and histopathological confirmation of resectable LAGC (cT2–4bNanyM0); (2) MSI-H or dMMR status prospectively determined by immunohistochemistry (IHC), polymerase chain reaction (PCR) or next-generation sequencing (NGS)-based testing on diagnostic biopsy tissue; (3) receipt of at least one cycle of neoadjuvant therapy—either immunotherapy alone, immunotherapy combined with chemotherapy, or chemotherapy alone; (4) gastrectomy with adequate lymph node dissection. Exclusion criteria included: (1) diagnosis of early gastric cancer (cT1N0M0) or confirmed distant metastasis (M1); (2) upfront surgical resection without prior neoadjuvant therapy; (3) history of another primary malignancy within the preceding five years; and (4) absence of essential clinical or pathological data required for analysis.

### 2.3. Treatments

Patients in the neoadjuvant immunotherapy-based treatment group (group A) received either PD-(L)1 inhibitor monotherapy or PD-(L)1 inhibitor combined with chemotherapy regimens. Patients in the chemotherapy-only group (group B) received neoadjuvant chemotherapy alone. Tumor response was assessed after completion of neoadjuvant therapy using standardized imaging and endoscopic criteria, followed by multidisciplinary tumor board review to determine resectability. Patients deemed candidates for R0 resection underwent standardized gastrectomy with D2 lymphadenectomy in accordance with the Japanese Gastric Cancer Treatment Guidelines [15]. Adjuvant therapy was administered postoperatively according to final pathologic staging and risk stratification. The PD-(L)1 blockade used included one of the following regimens: nivolumab (3 mg/kg iv drip, every two weeks), pembrolizumab (200 mg iv drip, every three weeks), sintilimab (200 mg iv drip, every three weeks), camrelizumab (200 mg iv drip, every three weeks), and tislelizumab (200 mg iv drip, every three weeks). The chemotherapy regimens included S-1, SOX, CAPOX, and FOLFOX (5-Fluorouracil, Leucovorin, and Oxaliplatin) regimens.

### 2.4. Assessments

The MMR status was evaluated using IHC staining. Primary antibodies against MSH2, MSH6, MLH1, and PMS2 were used for IHC staining of mismatch repair-related proteins. Generally, the absence of one or more of these proteins was considered the dMMR status. The status of MSI was evaluated using quantitative PCR or NGS by detecting the amplification of five MSI loci: BAT25, BAT26, D5S346, D17S250, and D2S123. MSI-H status was assigned when instability was detected in ≥2 of these loci.

Baseline and post-neoadjuvant tumor assessment was performed using contrast-enhanced computed tomography (CT) and endoscopic ultrasound (EUS), with treatment response evaluated per the Response Evaluation Criteria in Solid Tumors (RECIST) version 1.1 [16]. Surgical specimens—including primary tumor and regional lymph nodes—were staged according to the eighth edition of the American Joint Committee on Cancer (AJCC) Gastric Cancer Staging System. Pathologic tumor regression grade (TRG) was assessed using the AJCC-recommended four-tier classification: TRG0 (no viable cancer cells), TRG1 (single cells or small clusters of residual tumor cells), TRG2 (residual tumor masses with prominent fibrosis), and TRG3 (minimal or no regression; predominant viable tumor) [17]. All pathological evaluations were conducted independently by two board-certified gastrointestinal pathologists, with discrepancies resolved by consensus review.

### 2.5. Endpoint Evaluation

The primary endpoints were pCR, defined as TRG 0; MPR, defined as TRG0/1; EFS, defined as the period from treatment to any one of the following three events: unable to proceed with surgery due to disease progression, local or distant relapsed disease, or death caused by any reason; and OS, defined as the period from treatment to death caused by any reason. Secondary endpoints included surgical outcomes, and treatment-related adverse events (TRAEs). Adverse events were graded according to the Common Terminology Criteria for Adverse Events 5.0 (CTCAE 5.0) [18].

### 2.6. Follow-Up

After treatment, patients were recommended follow-up every 3 to 6 months for the first 2 years and every 6 to 12 months from years 3 to 5 by outpatient visits and/or telephone contact. Routine assessments included physical examination, imaging studies (contrast-enhanced CT, upper gastrointestinal endoscopy), and laboratory tests. Positron emission tomography–CT would be used when necessary. The date of the last follow-up was 1 March 2026.

### 2.7. Statistical Analysis

Continuous variables are presented as the median and range and were compared using the *t*-test if normally distributed or the Wilcoxon rank-sum test if nonnormally distributed; categorical and ordinal variables are presented as numbers and percentages and were compared using the Pearson chi-square test, Fisher’s exact test or Wilcoxon rank-sum test, as appropriate. Survival was estimated using Kaplan–Meier curves and compared with the log-rank test; hazard ratios (HRs) with 95% confidence intervals (CIs) were derived from Cox proportional hazards models. All statistical tests were two-sided. A *p*-value < 0.05 was considered statistically significant. All of these statistical analyses were performed using SPSS software version 23.0 (IBM, Armonk, NY, USA) and R software version 4.3.2.

### 2.8. Meta-Analysis

The study protocol has been prospectively registered with PROSPERO (CRD420261346671) and followed the Preferred Reporting Items for Systematic Reviews and Meta-Analyses (PRISMA) guidelines [19]. Two independent authors (PHY and CY) comprehensively searched the online databases of PubMed, Embase, Cochrane Library and Web of Science for potential studies from inception to 1 March 2026. The detailed search strategies for each database are presented in Table S1. Additionally, the gray literature from Google Scholar and Research Square was searched for potentially eligible reports. Inclusion criteria were as follows: (1) studies comparing group A with group B in LAGC patients who were confirmed as having dMMR/MSI-H status and who received gastrectomy; (2) studies reporting at least one of the following interesting outcomes: pCR, MPR, and survival outcomes; (3) for duplicated studies, only the study that included the most cases was included. Exclusion criteria included reviews, case reports, and letters. Data extraction was performed using a predefined form that captured study characteristics (e.g., publication year, region, sample size, study design), clinicopathological features (e.g., clinical stage, neoadjuvant regimen), and interesting outcomes. The risk of bias was assessed using the Risk of Bias in Non-Randomized Studies-of Interventions tool (ROBINS-I) for cohort studies [20].

All pooled analyses were conducted using R Software version 4.3.2. Heterogeneity between studies was evaluated using the I^2^ statistic; an I^2^ value greater than 50% indicated substantial heterogeneity. For dichotomous variables, the pooled risk ratios (RRs) were calculated using the Hartung–Knapp–Sidik–Jonkman (HKSJ) random-effects model to reduce the risk of false positives. Funnel plot with Begg’s test was performed to test publication bias. Trim-and-fill analysis was conducted when the publication bias test was significant (*p* < 0.10). For survival outcomes, the IPD survival data were extracted from the published swimming plot, as it displayed the detailed individual survival information, or from published survival curves according to the methods reported by Guyot et al. [21]. After that, the Kaplan–Meier method was applied to create the reconstructed survival curves. The Cox proportional hazards model based on the shared frailty model was applied to compute the random-effects HR along with its corresponding 95% CI of the two groups [22]. Statistical significance was defined as a two-tailed *p*-value < 0.05.

## 3. Results

### 3.1. Patient Characteristics and Neoadjuvant Treatments

From 2020 to 2025, a total of 42 dMMR/MSI-H gastric cancer patients were diagnosed at our center. After applying the eligibility criteria, 24 patients were enrolled, including 14 patients in group A and 10 in group B. Among patients in group A, 10 patients underwent ICI plus chemotherapy, and three underwent ICI alone. Chemotherapy regimens in this group included SOX, CAPOX, FOLFOX, and S-1. Among the patients in group B, eight patients received SOX, one received FOLFOX, and one received CAPOX. As shown in Table 1, baseline variables were all comparable between the two groups, including age, sex, BMI, ECOG performance status score, tumor location, tumor size, clinical TNM stage, MSI status, dMMR status, and HER2 status (all *p*-values > 0.05).

### 3.2. Surgical and Pathological Response Outcomes

Following neoadjuvant therapy, all patients were deemed eligible for surgical intervention. Finally, 14 patients in group A and 10 in group B underwent laparoscopic distal or total gastrectomy with D2 lymphadenectomy. R0 resection was achieved in all of these patients. Compared with patients in group B, those in group A showed comparable numbers of harvested lymph nodes and postoperative complication rates (Table 2).

**Figure 2:**
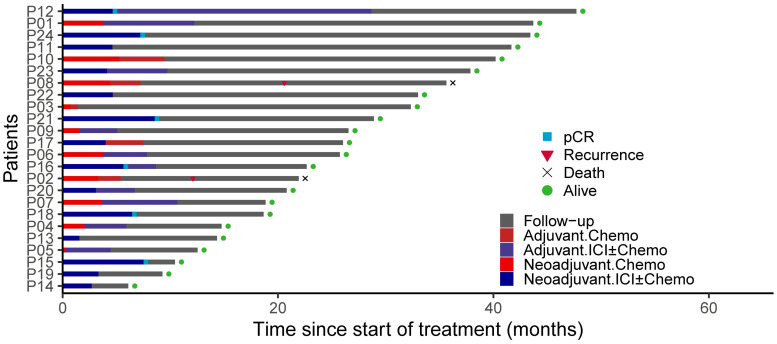
Swimming plot of the treatment process and duration of tumor response of the included patients.

As shown in Figure 2 and Table 3, among the 14 patients in group A, six (42.9%) achieved TRG 0 and six (42.9%) achieved TRG 1. Subgroup analysis revealed that, among the three patients who received ICI monotherapy, one (33.3%) achieved TRG 0 and one (33.3%) achieved TRG 1; among the 11 patients who received ICI combined with chemotherapy, five (45.5%) achieved TRG 0 and five (45.5%) achieved TRG 1. No statistically significant difference in TRG distribution was observed between these two subgroups (*p* = 0.555). In contrast, among the 10 patients in group B, none achieved TRG 0, and only two (20.0%) achieved TRG 1. Overall, the pCR rate (42.9% vs. 0%; *p* = 0.024) and MPR rate (85.7% vs. 20.0%; *p* = 0.003) appeared to be higher in group A than in group B.

### 3.3. Survival Outcomes

At the time of data cutoff, the median follow-up time was 24.4 months (range, 6.1–47.7 months) and 26.6 months (range, 12.5–43.7 months) for group A and group B, respectively. Median OS and EFS time were not reached in either group. As shown in Figure 2, two patients in group B (P02 and P08) experienced disease relapse followed by death, with EFS times of 12.3 and 20.8 months and OS times of 21.9 and 35.6 months, respectively. No events—including relapse, progression, or death—were observed in group A during the entire follow-up period, regardless of treatment regimen (ICI monotherapy or ICI combined with chemotherapy). Kaplan–Meier estimates of OS and EFS are presented in Figure S1. Compared with group B, group A seemed to exhibit a trend toward improved OS (log-rank test, *p* = 0.12) and EFS (log-rank test, *p* = 0.10). HRs for OS and EFS could not be estimated due to insufficient events in group A.

### 3.4. Safety

During neoadjuvant treatment, any-grade TRAEs occurred in 14 (100%) patients in group A and in 10 (100%) in group B. The common TRAEs for both groups included nausea, vomiting, hepatitis, myelosuppression, and fatigue. For patients in the two groups, no grade ≥ 3 event or treatment discontinuation occurred (Table 4).

### 3.5. Meta-Analysis

A systematic database search yielded 1682 records. Following rigorous screening of titles, abstracts, and full-text articles against prespecified eligibility criteria, eight studies [7,13,14,23,24,25,26,27] were included (Figure S2 and Table 5). Publication years ranged from 2023 to 2025. Among the included studies: one was a retrospective cohort study reporting primary outcomes specifically in patients with dMMR/MSI-H LAGC [14]; one was a conference abstract that provided analyzable pathological response data despite lacking full-text publication [26]; one was an IPD meta-analysis [7] integrating results from the PROSECCO study, NEONIPIGA trial, and INFINITY trial; and the remaining five studies [13,23,24,25,27] reported outcomes for dMMR/MSI-H LAGC patients as either prespecified (n = 2) or exploratory (n = 3) subgroup analyses within unselected LAGC populations. Ultimately, in addition to our cohort, a total of 337 dMMR/MSI-H LAGC patients who underwent gastrectomy were included—183 assigned to group A and 154 to group B. These studies were evaluated using the ROBINS-I tool: three studies (33.3%) were judged to be at low overall risk of bias, whereas six studies (66.7%) were assessed as having a high overall risk of bias, primarily attributable to serious concerns regarding confounding, missing outcome data, or selective reporting of results (Table S2).

**Figure 3:**
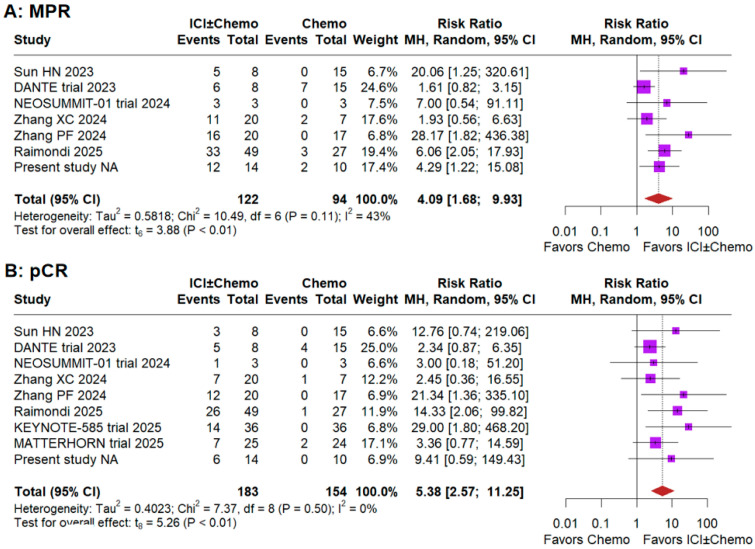
Forest plots of pathological response outcomes between the two groups. (**A**) MPR (major pathological response) [7,14,23,24,25,26] and (**B**) pCR (pathological complete response) [7,13,14,23,24,25,26,27].

As shown in Figure 3, seven studies comprising 216 surgical patients reported MPR; the pooled RR was 4.09 (95% CI: 1.68–9.93; *p* < 0.01; I^2^ = 43%). For pCR, nine studies including 337 surgical patients were analyzed; the pooled RR was 5.38 (95% CI: 2.57–11.25; *p* < 0.01; I^2^ = 0%). Sensitivity analyses restricted to the three studies (n = 127) assessed as low risk of bias demonstrated that patients in group A had a 3.80-fold higher pCR rate and a 6.50-fold higher MPR rate compared with those in group B (Figure S3); however, these differences did not reach statistical significance, likely attributable to limited statistical power (*p* = 0.06 for pCR; *p* = 0.08 for MPR). Additionally, we conducted exploratory subgroup analyses comparing pathological response outcomes across distinct immunotherapy regimens—including ICI monotherapy, ICI combined with chemotherapy, dual ICIs, and mixed regimens—versus chemotherapy alone. As shown in Figure S4, no statistically significant heterogeneity was observed among these regimen-based subgroups (*p* = 0.29 for MPR; *p* = 0.46 for pCR). Finally, assessment of publication bias for both MPR and pCR—using funnel plots supplemented by Begg’s test—revealed no evidence of asymmetry (MPR: *p* = 0.133; pCR: *p* = 0.175; Figure S5).

**Figure 4:**
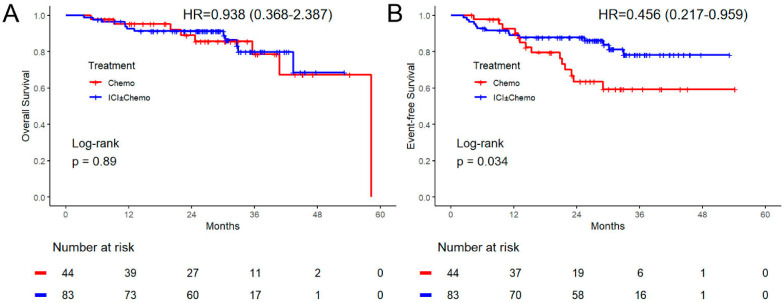
Reconstructed Kaplan–Meier survival curves between the two groups. (**A**) Overall survival; (**B**) event-free survival.

For survival outcomes, three baseline-balanced studies—encompassing a total of 127 patients and reporting both OS and EFS—were included (83 in group A; 44 in group B). Reconstructed survival curves, alongside visual comparisons with the originally published survival curves or swimming plot, are presented in Table S3. The reconstructed estimates were highly concordant with the published survival information across all studies, with negligible discrepancies. Median follow-up duration was 29.2 months (range, 3.5–53.1 months) in group A and 28.1 months (range, 4.8–58.3 months) in group B. Pooled analysis of OS based on reconstructed Kaplan–Meier curves indicated no statistically significant difference between group A and group B (HR = 0.938; 95% CI: 0.368–2.378; *p* = 0.890; Figure 4A). In contrast, group A seemed to demonstrate significantly improved EFS compared with group B (HR = 0.456; 95% CI: 0.217–0.959; *p* = 0.034; Figure 4B). Furthermore, exploratory subgroup analyses were conducted to compare survival outcomes across distinct immunotherapy regimens—including ICI monotherapy, ICI combined with chemotherapy, dual ICIs, and mixed regimens—versus chemotherapy alone. As shown in Figure S6, no statistically significant differences in OS (log-rank test, *p* = 0.37) or EFS (log-rank test, *p* = 0.064) were observed among these regimen-based subgroups. At 24 months, the OS rates were 100%, 100%, 85.5%, and 85.6% for patients receiving ICI monotherapy, ICI combined with chemotherapy, dual ICIs, and chemotherapy alone, respectively; the corresponding 24-month EFS rates were 100%, 90.9%, 81.4%, and 63.5%.

## 4. Discussion

Real-world evidence from our small-sample study suggested that, among surgical candidates with dMMR/MSI-H LAGC, neoadjuvant PD-(L)1 inhibitors, used either alone or in combination with chemotherapy, may confer certain advantages. Specifically, there appeared to be an improvement in MPR and pCR rates. Additionally, a trend towards the prolongation of EFS and OS was observed when compared with neoadjuvant chemotherapy alone. Simultaneously, no new safety concerns related to surgical outcomes and TRAEs were identified in the immunotherapy-based treatment group. To gain a more comprehensive understanding of the potential benefits of neoadjuvant immunotherapy-based regimens for these patients, we carried out a meta-analysis with data from our single-center cohort and eight other relevant studies. By pooling the data of 337 patients, it was found that immunotherapy-based regimens were associated with higher MPR and pCR rates; however, sensitivity analyses restricted to studies at low risk of bias did not yield significant differences. For long-term outcomes, immunotherapy-based treatment showed potential for improving EFS. However, the evidence for OS benefit remained inconclusive at this stage.

Substantial clinical and preclinical evidence robustly supports the enhanced sensitivity of gastric cancer patients with dMMR/MSI-H status to ICIs [10,28]. This improved therapeutic response is mechanistically underpinned by elevated tumor mutational burden and the resultant expansion of immunogenic neoantigens—features that collectively foster a more immunoreactive tumor microenvironment [29]. After treatment, immunotherapy could induce a more pronounced remodeling of the tumor microenvironment relative to chemotherapy in these patients. These changes mainly encompass the following biological dimensions: (1) changes in immune cells: enhanced activation of dendritic cells, increased recruitment and stimulation of CD8^+^ T cells, decreased population of immunosuppressive regulatory T cells, and polarization of tumor-associated macrophages to the M1 phenotype; (2) further upregulated expression of immune checkpoint molecules (PD-L1, CTLA-4, LAG3, TIM3); (3) increased release of tumor neoantigens; (4) normalization of tumor blood vessels and remodeling of the extracellular matrix [30,31]. In summary, these changes in the tumor microenvironment usually contribute to a favorable response to immunotherapy in this subset of patients. However, limited by the retrospective nature of our study, we were unable to perform dynamic monitoring on the changes of these biomarkers. Further, reflected in clinical practice, a recent meta-analysis [6]—integrating data from 20 studies involving 396 patients with dMMR/MSI-H LAGC—reported pooled pCR and MPR rates of 41.9% and 64.2%, respectively, almost consistent with our updated analysis. In contrast, our study indicated that neoadjuvant chemotherapy yielded substantially lower pCR (5.2%) and MPR (14.9%) rates, reinforcing the therapeutic advantage of ICI-based neoadjuvant regimens in this molecularly defined population. However, it should be noted that the robustness of subgroup and sensitivity analyses in our study was compromised by a relatively small sample size and suboptimal quality across included studies. Consequently, the current evidence still remains insufficient to establish the superiority of immune-based neoadjuvant therapy over chemotherapy in these patients.

The most important criterion for the application of neoadjuvant immunotherapy strategies is their ability to confer significant survival benefits. In unselected LAGC patients, the latest meta-analysis [32] confirmed that neoadjuvant immunotherapy combined with chemotherapy can significantly improve OS and recurrence-free survival in patients with LAGC, suggesting that neoadjuvant immunotherapy has shown potential for survival benefits in those patients. However, it remains unclear whether the dMMR/MSI-H subgroup constitutes the primary beneficiary population, owing to the absence of subgroup-level data. Moreover, subgroup analyses from two large-scale randomized controlled trials—KEYNOTE-585 [27] and MATTERHORN [13]—failed to demonstrate that neoadjuvant immunotherapy regimens confer a statistically significant survival benefit in those patients. Due to the relatively low proportion of dMMR/MSI-H patients and their significant response to ICIs, conducting large-scale randomized controlled trials seems infeasible and unethical. Given this, we conducted an IPD survival analysis by integrating data from our center and the currently available literature (all of these studies were regarded as having a low risk of bias). Although our study found no significant OS benefit with neoadjuvant immunotherapy-based treatment, it suggests a possible EFS improvement in this subgroup population (with numerically consistent results in different types of immunotherapy regimens). The benefit to EFS may mainly be related to the improvement in pathological response results. However, this finding is limited by short follow-up and small sample size—factors that reduce confidence in the EFS signal. Meanwhile, OS is a complex and time-intensive endpoint. In clinical practice, substantial crossover to immunotherapy during the adjuvant phase—particularly among patients initially assigned to neoadjuvant chemotherapy—may dilute or obscure the true treatment effect of neoadjuvant immunotherapy. For instance, in our neoadjuvant chemotherapy cohort, 60% (6/10) of patients received adjuvant immunotherapy-based therapy. Furthermore, the current clinical guidelines recommend immunotherapy in the first or the later lines for patients with dMMR/MSI-H gastric cancer who are immunotherapy-naïve and subsequently develop recurrent or metastatic disease—a strategy strongly supported by robust evidence demonstrating significant OS prolongation in this molecularly defined population [33,34].

Regarding the safety profile, most clinical studies suggest that neoadjuvant immunotherapy-based regimens do not meaningfully impair perioperative outcomes or increase the incidence of TRAEs in LAGC patients. Notably, phase III trials—such as MATTERHORN [13] and KEYNOTE-585 [27]—reported no statistically significant differences between immunotherapy-containing and chemotherapy-only arms in terms of surgical complication rates or TRAEs. Real-world evidence from Wang et al. [35] further supported this finding, demonstrating that gastrectomy with D2 or D2+ lymphadenectomy following preoperative immunotherapy plus chemotherapy yielded short-term oncological outcomes comparable to those achieved with preoperative chemotherapy alone. In line with these studies, the immunotherapy group in our cohort study exhibited no statistically significant differences for any perioperative outcomes or TRAEs.

The present study has several limitations that should be acknowledged. First, consistent with these included studies, our single-center retrospective study had a very limited sample size. Consequently, multivariate regression analysis or propensity score matching analysis could not be performed to adjust the potential confounding covariates on the outcomes. Second, several included studies contributed data from post hoc subgroup analyses of randomized controlled trials or from subgroup analyses of retrospective studies—contexts in which baseline comparability between groups cannot be assured. Consequently, the inclusion of these high-risk-of-bias studies compromises the validity of our pooled estimates. Moreover, sensitivity analyses excluding such studies failed to yield statistically significant results, largely attributable to the substantially reduced sample size Third, the therapeutic regimens of the intervention groups in most studies exhibited considerable heterogeneity, encompassing ICI monotherapy, ICI combined with chemotherapy, and dual ICIs. Although the exploratory analysis we conducted comparing these regimens with chemotherapy alone in terms of pathological response and long-term survival outcomes did not yield a conclusion of statistically significant difference, the pooled inclusion of these regimens still increased the difficulty of interpreting the results. Finally, for the most clinically important endpoint—long-term survival—only three studies with adequately balanced baseline characteristics reported EFS and OS curves. The limited sample size across these studies constrained statistical power, while their relatively short follow-up durations yielded few events in both comparison groups, resulting in immature survival curves.

## 5. Conclusions

In resectable dMMR/MSI-H LAGC patients, our study found that neoadjuvant immunotherapy-based regimens did not appear to improve OS compared with chemotherapy alone but may be associated with higher MPR, pCR, and EFS rates while maintaining a generally comparable safety profile. These preliminary observations hint at the potential therapeutic value of neoadjuvant immunotherapy-based treatment in this patient population. However, further high-quality prospective studies with sufficient statistical power are still required to validate these findings.

## Figures and Tables

**Table 1:** Clinicopathologic features of included patients.

Variables		Group A (n = 14)	Group B (n = 10)	*p*-Value
Median age, years, (range)		61.5 (53–78)	57 (47–69)	0.069
Sex				
	Male	10 (71.4%)	6 (60%)	0.673
	Female	4 (28.6%)	4 (40%)	
ECOG PS				0.977
	0	6 (42.9%)	4 (40%)	
	1	7 (50%)	6 (60%)	
	2	1 (7.1%)	0 (0%)	
Median BMI, kg/m^2^ (range)		21.9 (19.2–23.1)	23.1 (18.7–26.5)	0.111
Tumor location				0.886
	GEJA	1 (7.1%)	1 (10%)	
	Upper third	2 (14.3%)	0 (0%)	
	Middle third	4 (28.6%)	4 (40%)	
	Lower third	7 (50%)	5 (50%)	
Median tumor size, cm (range)		5.4 (3.0–9.6)	4.9 (3.0–7.3)	0.221
Tumor differentiation				1.000
	Well differentiated	0 (0%)	0 (0%)	
	Moderate	3 (21.4%)	2 (20%)	
	Poor	11 (78.6%)	8 (80%)	
Clinical stage				0.931
	II	1 (7.1%)	1 (10%)	
	III	11 (78.6%)	7 (70%)	
	IVa	2 (14.3%)	2 (20%)	
Loss of dMMR protein (IHC)				1.000
	MLH1 and/or PMS2	13 (92.9%)	9 (90%)	
	MSH2 and/or MSH6	1 (7.1%)	1 (10%)	
MSI-H test (PCR or NGS)				0.472
	MSI-H	8 (57.2%)	8 (80%)	
	MSS	3 (21.4%)	0 (0%)	
	Unknown	3 (21.4%)	2 (20%)	
HER2 status				1.000
	0	12 (85.7%)	9 (90%)	
	1+ OR 2+ (FISH−)	1 (7.1%)	1 (10%)	
	2+ (FISH+) OR 3+	1 (7.1%)	0 (0%)	

ECOG PS: Eastern Cooperative Oncology Group Performance Status Score; BMI: body mass index; GEJA: adenocarcinoma of the gastroesophageal junction; dMMR: mismatch repair deficiency; MSI-H: high microsatellite instability; MSS: microsatellite stable status; IHC: immunohistochemistry; PCR: polymerase chain reaction; NGS: next-generation sequencing; FISH: fluorescence immunohistochemical in situ hybridization.

**Table 2:** Comparison of surgical outcomes according to the treatment groups.

Variables	Group A (n = 14)	Group B (n = 10)	*p*-Value
Laparoscopic gastrectomy	14 (100%)	10 (100%)	1.000
Gastrectomy extent			1.000
Distal gastrectomy	12 (85.7%)	8 (80%)	
Total gastrectomy	2 (14.3%)	2 (20%)	
R0 resection	14 (100%)	10 (100%)	1.000
Lymph nodes retrieved	34 (27–58)	35 (20–44)	0.381
Postoperative complications	5 (35.7%)	2 (20%)	0.653
Clavien–Dindo grade I–II	3 (21.4%)	2 (20%)	1.000
Clavien–Dindo grade III–IV	2 (14.3%)	0 (0%)	0.493
Abdominal infection	1 (7.1%)	0 (0%)	1.000
Ileus	2 (14.3%)	0 (0%)	0.493
Pulmonary embolism	0 (0%)	1 (10%)	0.417
Pneumonia	2 (14.3%)	1 (10%)	1.000

**Table 3:** Comparison of pathologic response outcomes according to the treatment groups.

Variables	Group A (n = 14)	Group B (n = 10)	*p*-Value
pCR (ypT0N0)	6 (42.9%)	0 (0%)	0.024
MPR	12 (85.7%)	2 (20%)	0.003
ypT stage			0.001
ypT0	6 (42.9%)	0 (0%)	
ypT1	6 (42.9%)	2 (20%)	
ypT2	1 (7.1%)	2 (20%)	
ypT3	0 (0%)	1 (10%)	
ypT4	1 (7.1%)	5 (50%)	
ypN stage			0.055
ypN0	12 (85.7%)	5 (50%)	
ypN1	2 (14.3%)	4 (40%)	
ypN2	0 (0%)	1 (10%)	
Tumor regression grade (AJCC 8th edition)			0.001
TRG 0	6 (42.9%)	0 (0%)	
TRG 1	6 (42.9%)	2 (20%)	
TRG 2	2 (14.3%)	8 (80%)	
TRG 3	0 (0%)	0 (0%)	

pCR: pathologic complete response; MPR: major pathologic response; TRG: tumor regression grade.

**Table 4:** Comparison of treatment-related adverse events according to the treatment groups.

	Group A (n = 14)		Group B (n = 10)	
	Any Grade	Grade 3–4	Any Grade	Grade 3–4
Any TRAE (max/patients)	14 (100%)	0 (0%)	10 (100%)	0 (0%)
Any TRAE leading to discontinuation	0 (0%)	0 (0%)	0 (0%)	0 (0%)
Fatigue	6 (42.9%)	0 (0%)	2 (20%)	0 (0%)
Vomiting	12 (85.7%)	0 (0%)	10 (100%)	0 (0%)
Nausea	12 (85.7%)	0 (0%)	10 (100%)	0 (0%)
Hepatitis (increased AST/ALT)	6 (42.9%)	0 (0%)	4 (40%)	0 (0%)
Myelosuppression	12 (85.7%)	0 (0%)	4 (40%)	0 (0%)

TRAE: treatment-related adverse event; AST: aspartic transaminase; ALT: alanine transaminase.

**Table 5:** The basic characteristics of the included studies, including the present study.

Reference (Study Interval)	Country	Study Design	Sample Size (Group A/B)	cTNM Stage	Intervention Group	Regimens Used	Endpoints
Sun HN, 2023 [23] (2019–2023)	Russia	R:S (subgroup with unknown baseline)	23 (8/15)	cT2 - 4N0/1M0	ICI + Chemo	Pembrolizumab; Nivolumab; FLOT; FOLFIRINOX; XELOX; FOLFOX	MPR; pCR
DANTE trial, 2023 [24] (2018–2020)	Europe	RCT; M (prespecified subgroup)	23 (8/15)	≥cT2 or cN1	ICI + Chemo	Atezolizumab; FLOT	MPR; pCR
NEOSUMMIT-01 trial, 2024 [25] (2019–2022)	China	RCT; M (post hoc subgroup)	6 (3/3)	cT3-4aN+M0	ICI + Chemo	Toripalimab; SOX; XELOX	MPR; pCR
Zhang XC, 2024 [14] (2019–2023)	China	R:S (whole cohort with balanced baseline)	27 (20/7)	III–IVa	ICI + Chemo ICI monotherapy	Nivolumab; Pembrolizumab; Sintilimab; Camrelizumab; Tislelizumab; Serplulimab; Envafolimab; FLOT; SOX; mFLOT; mDOS	MPR; pCR; EFS; OS
Zhang PF, 2024 [26] (2018–2023)	China	R:S (whole cohort with unknown baseline)	37 (20/17)	NA	ICI + Chemo ICI monotherapy Dual ICIs	NA	MPR; pCR
Raimondi, 2025 [7] (2017–2022)	Europe and USA	IPD pooled analysis (with balanced baseline)	76 (49/27)	T2-4NanyM0	Dual ICIs	Tremelimumab plus Durvalumab; Nivolumab plus Ipilimumab; FLOT	MPR; pCR; EFS; OS
KEYNOTE-585 trial, 2025 [27] (2017–2021)	International	RCT; M (prespecified subgroup)	72 (36/36)	II–IVa	ICI + Chemo	Pembrolizumab; XP; FP; FLOT	pCR
MATTERHORN trial, 2025 [13] (2020–2022)	International	RCT; M (post hoc subgroup)	49 (25/24)	II–IVa	ICI + Chemo	Durvalumab; FLOT	pCR
Present study, (2020–2025)	China	R:S (whole cohort with balanced baseline)	24 (14/10)	II–IVa	ICI + Chemo ICI monotherapy	Nivolumab; Pembrolizumab; Sintilimab; Camrelizumab; Tislelizumab; S-1; SOX; CAPOX; FOLFOX	MPR; pCR; EFS; OS

R: retrospective study; RCT: randomized controlled trial; S: single center; M: multiple centers; ICI: immune checkpoint inhibitor; Chemo: chemotherapy; pCR: pathologic complete response; MPR: major pathologic response; OS: overall survival; EFS: event-free survival; NA: not available.

## Data Availability

We have already uploaded the required core data to the submission system. The remaining data presented in this study are available on request from the corresponding author. Due to institutional governance and privacy restrictions, the complete data are not publicly available.

## Supplementary Materials

The following supporting information can be downloaded at: https://www.mdpi.com/article/10.3390/cancers18122017/s1: Table S1. Search strategies of each included database. Table S2. Literature quality assessment using ROBINS-I tool. Table S3. Original and reconstructed survival information from the included studies. Figure S1. Kaplan-Meier curves for neoadjuvant immunotherapy ± chemotherapy versus chemotherapy alone in dMMR/MSI-H LAGC patients undergoing gastrectomy. A: overall survival; B: event-free survival. Figure S2. PRISMA flowchart of included studies. Figure S3. Sensitivity analyses of pathological response outcomes based on low-risk-of-bias studies. (A) MPR and (B) pCR. MPR: major pathological response; pCR: pathological complete response. Figure S4. Subgroup analyses of pathological response outcomes based on different immunotherapy regimens versus chemotherapy alone. (A) MPR and (B) pCR. MPR: major pathological response; pCR: pathological complete response. Figure S5. Funnel plots along with Begg’s tests assessing publication bias for (A) MPR and (B) pCR. MPR: major pathological response; pCR: pathological complete response. Figure S6. Kaplan–Meier curves for different immunotherapy regimens versus chemotherapy alone in dMMR/MSI-H LAGC patients undergoing gastrectomy. A: overall survival; B: event-free survival.

## Abbreviations

The following abbreviations are used in this manuscript:

LAGC	Locally advanced gastric cancer
dMMR	Mismatch repair deficiency
MSI-H	Microsatellite instability high
ICI	Immune checkpoint inhibitor
R0 resection	Radical resection
EFS	Event-free survival
OS	Overall survival
IPD	Individual patient data
IHC	Immunohistochemistry
PCR	Polymerase chain reaction
NGS	Next-generation sequencing
MPR	Major pathological response
cCR	Clinical complete response
pCR	Pathological complete response
CR	Complete response
NOM	Non-operative management
CT	Computed tomography
EUS	Endoscopic ultrasound
RECIST	Response evaluation criteria in solid tumors
AJCC	American Joint Committee on Cancer
TRG	Tumor regression grade
TRAE	Treatment-related adverse event
CTCAE	Common terminology criteria for adverse event
FOLFOX	5-fluorouracil, leucovorin, and oxaliplatin
FLOT	5-fluorouracil, leucovorin, oxaliplatin and docetaxel
SOX	S-1 and oxaliplatin
CAPOX	Capecitabine and oxaliplatin
HR	Hazard ratio
RR	Risk ratio
CI	Confidence interval
HKSJ	Hartung–Knapp–Sidik–Jonkman
ROBINS-I	Risk of bias in non-randomized studies of interventions tool
