# Supplementary material for: Neoadjuvant Immunotherapy-Based Treatment Versus Chemotherapy Alone in Resectable Locally Advanced dMMR/MSI-H Gastric Cancer: A Real-World Study with Meta-Analysis
Source: Cancers (Basel). 2026 Jun 22;18(12):2017. doi: 10.3390/cancers18122017 (PMC13297142; doi:10.3390/cancers18122017)
Supplement: Supplementary file 1 [file cancers-18-02017-s001.zip › cancers-4352028-supplementary.pdf]

Table S1. Search strategies of each included database.

Database	Search strategy	Number of studies
PubMed	(((gastric cancer OR gastric carcinoma OR stomach cancer OR stomach tumor OR gastroesophageal junction cancer) AND (Neoadjuvant OR Perioperative OR preoperative)) AND (immune checkpoint inhibitor OR PD-1 OR PD-L1 OR immunotherapy)) AND (Chemotherapy)<br>: searched in All Fields	604
Embase	(((gastric cancer OR gastric carcinoma OR stomach cancer OR stomach tumor OR gastroesophageal junction cancer) AND (Neoadjuvant OR Perioperative OR preoperative)) AND (immune checkpoint inhibitor OR PD-1 OR PD-L1 OR immunotherapy)) AND (Chemotherapy).mp.	380
Web of Science	#1 gastric cancer OR gastric carcinoma OR stomach cancer OR stomach tumor OR gastroesophageal junction cancer (All Fields)<br>#2 neoadjuvant OR perioperative OR preoperative (All Fields)<br>#3 immune checkpoint inhibitor OR PD-1 OR PD-L1 OR immunotherapy (All Fields)<br>#4 chemotherapy (All Fields)<br>#5 #1 and #2 and #3 and #4	543
Cochrane Library	#1 gastric cancer OR gastric carcinoma OR stomach cancer OR stomach tumor OR gastroesophageal junction cancer [All Text]<br>#2 neoadjuvant OR perioperative OR preoperative [All Text]<br>#3 immune checkpoint inhibitor OR PD-1 OR PD-L1 OR immunotherapy [All Text]<br>#4 chemotherapy [All Text]<br>#6 #1 and #2 and #3 and #4	154

Table S2. Literature quality assessment using ROBINS-I tool.

**Study**	**Confounding**	**Selection of participants**	**Classification of interventions**	**Deviation from intended interventions**	**Missing data**	**Measurement of outcomes**	**Selection of the reported result**	**Overall**
Sun HN, 2023	Severe	Low	Low	Low	Severe	Low	Severe	Severe
DANTE trial,2023	Low	Low	Low	Low	Severe	Low	Severe	Severe
NEOSUMMIT-01 trial, 2024	Moderate	Low	Low	Low	Severe	Low	Severe	Severe
Zhang XC,2024	Low	Low	Low	Low	Low	Low	Low	Low
Zhang PF,2024	Moderate	Low	Low	Low	Severe	Moderate	Severe	Severe
Raimondi,2025	Low	Low	Low	Low	Low	Low	Low	Low
KEYNOTE-585 trial,2025	Low	Low	Low	Low	Severe	Low	Severe	Severe
MATTERHORN trial,2025	Moderate	Low	Low	Low	Severe	Low	Severe	Severe
Present study	Low	Low	Low	Low	Low	Low	Low	Low

Table S3. Original and reconstructed survival information from the included studies.

Reference	Primary survival curve or swimming plot	Reconstructed survival curve
Raimondi,2025 (Event-free survival)	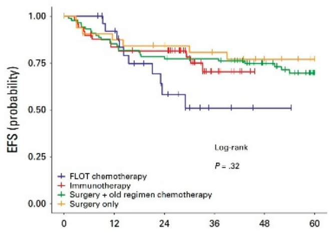	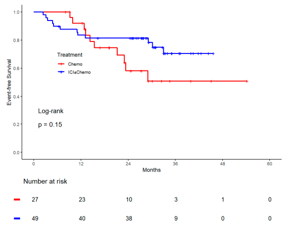
Raimondi,2025 (Overall survival)	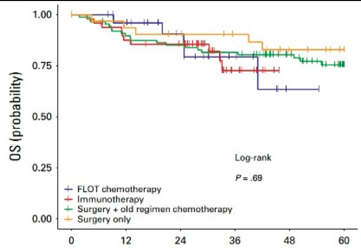	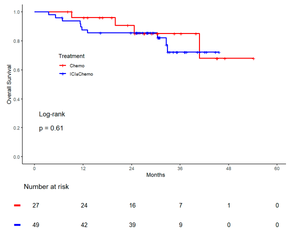
Zhang XC,2024 (Event-free survival)	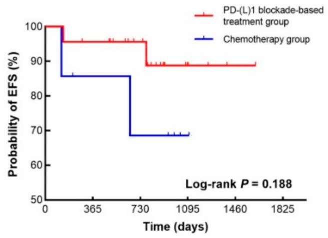	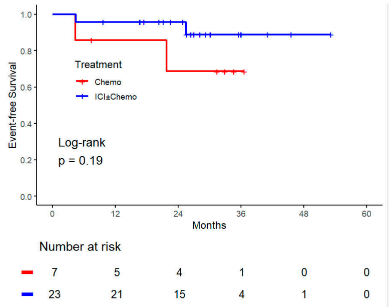
Zhang XC,2024 (Overall survival)	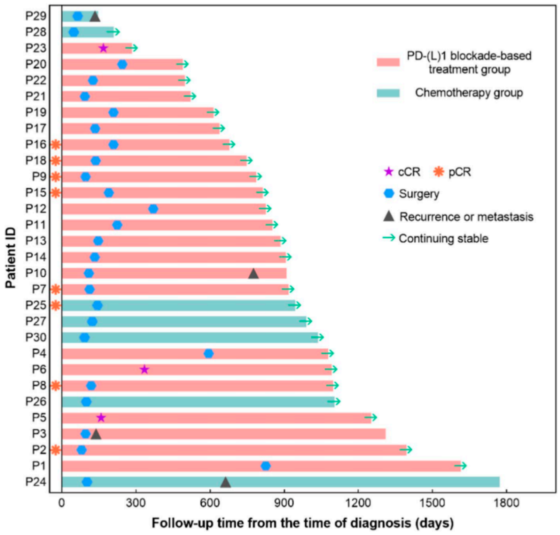	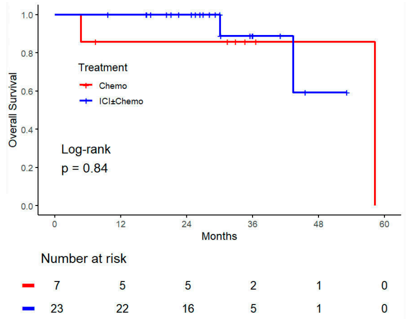

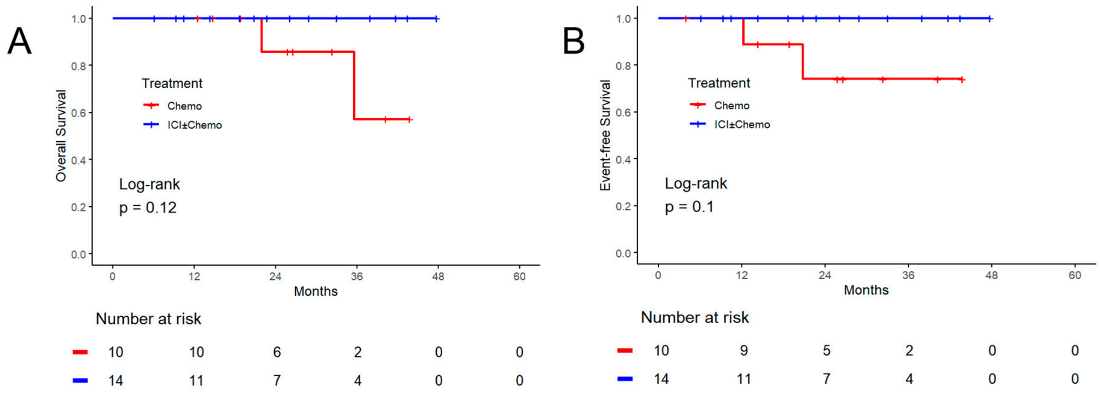

Figure S1. Kaplan-Meier curves for neoadjuvant immunotherapy ± chemotherapy versus chemotherapy alone in dMMR/MSI-H LAGC patients undergoing gastrectomy. A: overall survival; B: event-free survival.

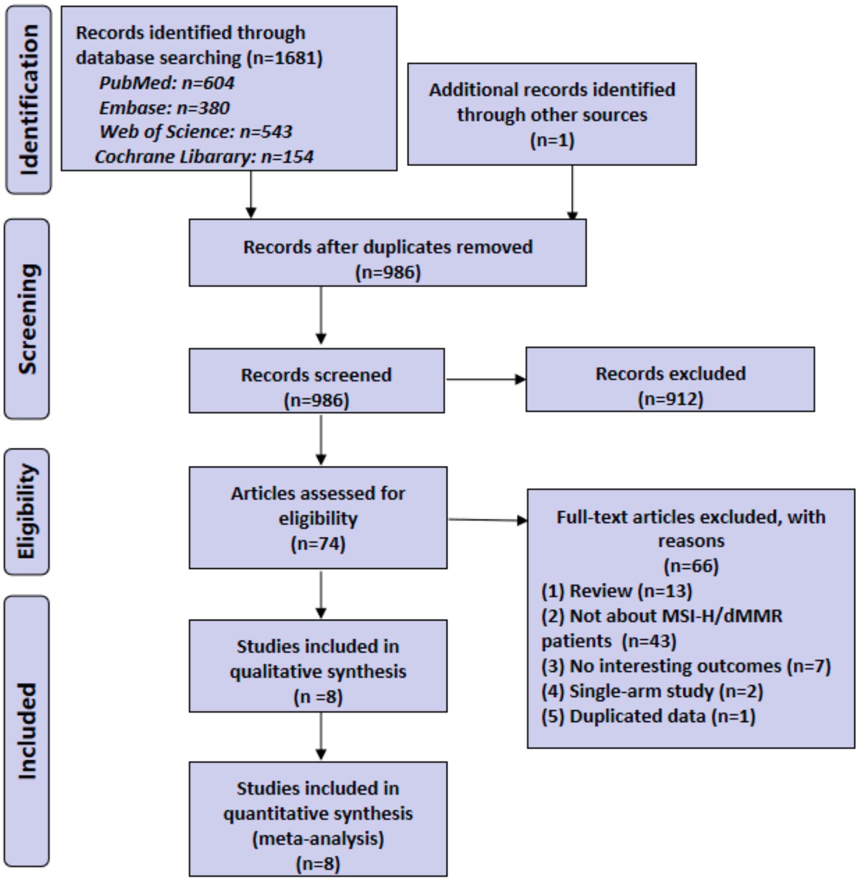

Figure S2. PRISMA flowchart of included studies.

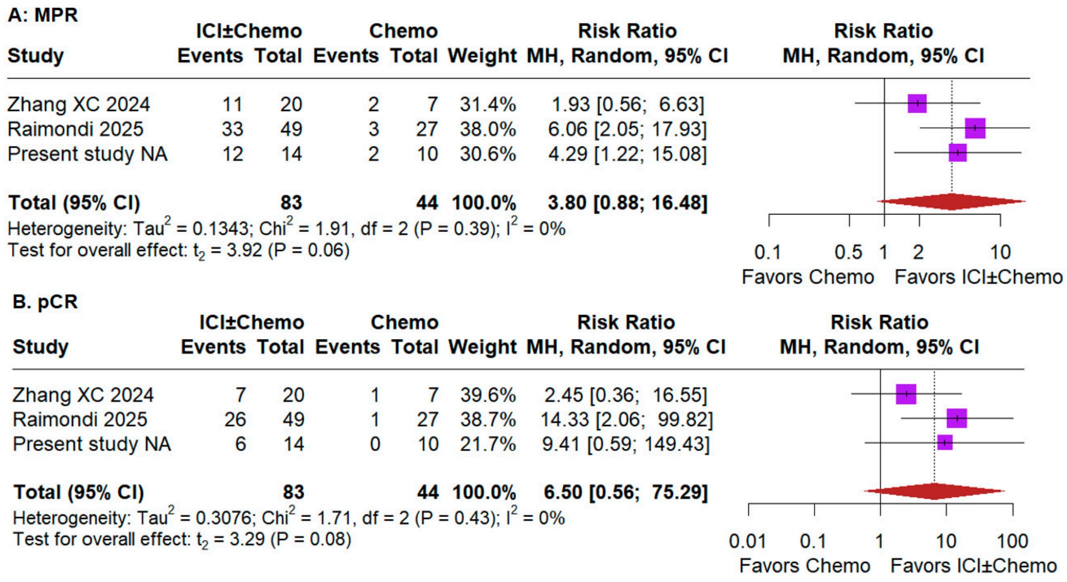

Figure S3. Sensitivity analyses of pathological response outcomes based on low-risk-of-bias studies. (A) MPR and (B) pCR. MPR: Major pathological response; pCR: Pathological complete response.

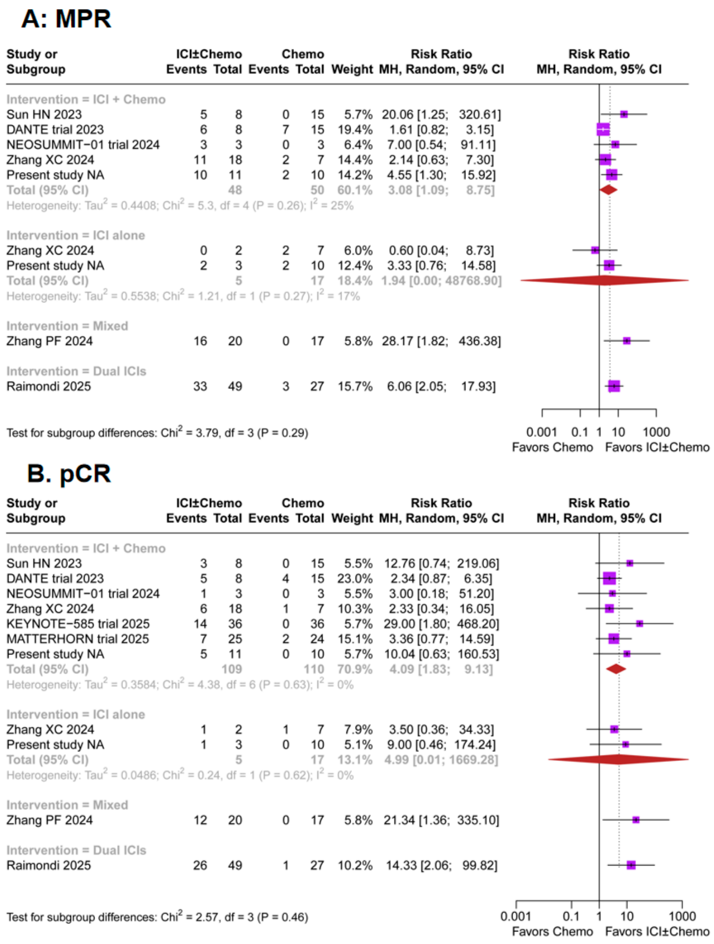

Figure S4. Subgroup analyses of pathological response outcomes based on different immunotherapy regimens versus chemotherapy alone. (A) MPR and (B) pCR. MPR: Major pathological response; pCR: Pathological complete response.

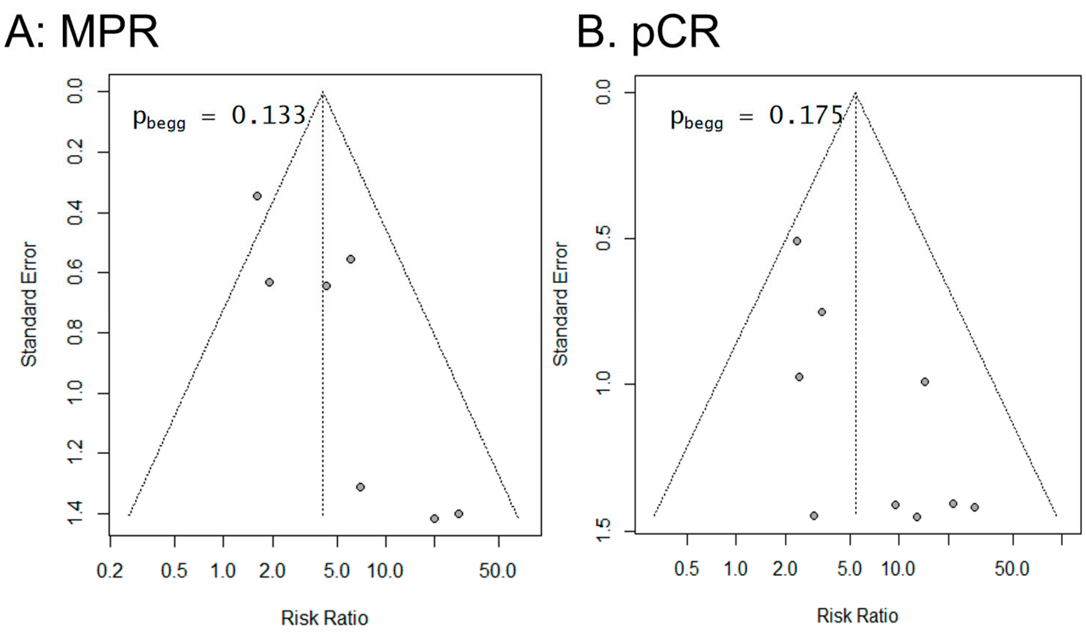

Figure S5. Funnel plots along with Begg's tests assessing publication bias for (A) MPR and (B) pCR. MPR: Major pathological response; pCR: Pathological complete response.

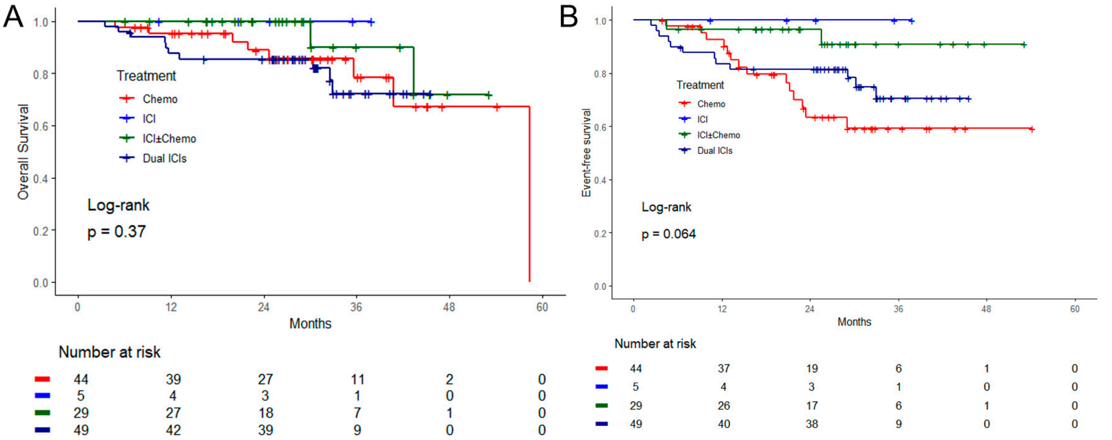

Figure S6. Kaplan-Meier curves for different immunotherapy regimens versus chemotherapy alone in dMMR/MSI-H LAGC patients undergoing gastrectomy. A: overall survival; B: event-free survival.
